# Effects of Faces and Voices on the Encoding of Biographic Information

**DOI:** 10.3390/brainsci12121716

**Published:** 2022-12-15

**Authors:** Sarah Fransson, Sherryse Corrow, Shanna Yeung, Heidi Schaefer, Jason J. S. Barton

**Affiliations:** 1 Faculty of Medicine, Linköping University, 581 83 Linköping, Sweden; 2 Human Vision and Eye Movement Laboratory, Departments of Medicine (Neurology), Ophthalmology and Visual Sciences, Psychology, University of British Columbia, Vanacouver, BC V5Z 3N9, Canada; 3 Department of Psychology, Bethel University, St. Paul, MN 55112, USA

**Keywords:** semantic, memory, learning, person, facilitation

## Abstract

There are multiple forms of knowledge about people. Whether diverse person-related data interact is of interest regarding the more general issue of integration of multi-source information about the world. Our goal was to examine whether perception of a person’s face or voice enhanced the encoding of their biographic data. We performed three experiments. In the first experiment, subjects learned the biographic data of a character with or without a video clip of their face. In the second experiment, they learned the character’s data with an audio clip of either a generic narrator’s voice or the character’s voice relating the same biographic information. In the third experiment, an audiovisual clip of both the face and voice of either a generic narrator or the character accompanied the learning of biographic data. After learning, a test phase presented biographic data alone, and subjects were tested first for familiarity and second for matching of biographic data to the name. The results showed equivalent learning of biographic data across all three experiments, and none showed evidence that a character’s face or voice enhanced the learning of biographic information. We conclude that the simultaneous processing of perceptual representations of people may not modulate the encoding of biographic data.

## 1. Introduction

Recognizing people is an important human social skill. While face recognition has been one of the most studied aspects of person recognition, there are many other cues that can be used to identify people [1,2,3,4]. These range from other visual cues, such as body shape, gait and handwriting, to non-visual sensory cues such as voice, and to semantic information, such as name and biographic data. 

The cognitive operations that support these diverse recognition processes remain topics of study. Earlier cognitive stage models [5] focused on face processing. These envisioned operations that proceed from early visual stages to more face-specific perceptual processing, followed by matching of the resulting percept to representations of previously seen faces in ‘face recognition units’. Successful matches then trigger access to semantic information in a more conceptual and amodal ‘person-identity node’. Subsequently, others have envisioned similar parallel processes for recognition through other non-facial cues [3,6,7], sometimes with modifications that take into account hemispheric specialization for different forms of information [8].

How these different cues, operations and processes interact and integrate is a topic of debate, not only for person recognition but also for semantic knowledge in general. Some argue for convergence of information in a central amodal semantic hub that binds and mediates access across modalities [9,10], while others suggest a ‘distributed-only’ view in which representations in different modalities interact directly, without the need for mediation by a central hub [11]. In more general terms, both of these concepts are examples of hierarchically shallow models, with the person hub representing a single convergence zone [12]. For person recognition, evidence for these models has been sought in studies of patients with cerebral lesions that assess whether patients can link information across modalities, although the quality and quantity of information varies widely across such reports [1,2].

The concept of multimodal integration has also led to a search for interactions between different sources of person identification in healthy subjects. Numerous studies have looked for face–voice interactions—for reviews, see Refs. [13,14]. However, there have been fewer reports looking for interactions involving semantic information, and much of the work that exists relates to names rather than to biographic data [1]. Hence, whether any interactions are general or specific to certain stimulus combinations is not known. Furthermore, how and where such interactions occur in the processing of a person’s information is not clear. Given the multiplicity of operations involved in identifying people, there are a number of potential sites for interaction.

First, in remembering information about people, interactions could occur independently in either the encoding or retrieval processes. While both likely operate on the same stored representations, they differ in other respects. Experiments with divided attention [15] suggest that encoding involves conscious controlled processing of perceptual experiences, while retrieval, while demanding of cognitive resources, may have a greater degree of automaticity, though this may vary with the type of retrieval demanded (e.g., free recall, cued recall, recognition, etc.). The differences between encoding and retrieval mean that we cannot assume that the presence of multimodal interactions in one process means that they will also be present in the other. 

Second, those different types of recall could vary in their potential to reveal multimodal interactions. Familiarity tasks have subjects respond as to whether they have encountered that information or stimulus before. In cognitive models, familiarity could arise from activation of either modality-specific recognition units—i.e., activating a face memory—or higher level amodal person identity nodes—i.e., recognizing the person whose face it is. On the other hand, identifying the stimulus or information as belonging to a specific person, as when naming them, involves a response that already requires information linkage at an amodal level. One could speculate that a response emanating from such an integrative stage would be more likely to show multimodal effects.

It is also possible that different types of interactions occur. One might expect a facilitative effect whereby the encoding of person information would be strengthened or reinforced by a second source of information being present during learning. In the case of two redundant sensory sources, such as the face and the voice in synchrony, audiovisual integration may contribute to this facilitation [16]. However, a second source of information may generate interference effects that impair the encoding of the first. On a general level, this could reflect a negative impact of dividing attention when attentional capacity is limited [15]. Indeed, such a detrimental effect has been reported as ‘face overshadowing’ in studies where voice learning has been impeded rather than enhanced by the presence of a face [17,18].

In this report, we performed three experiments that had the goal of determining whether the encoding of biographic data, a form of amodal, often verbal semantic information about people, is enhanced by the concurrent presence of the sensory cues of a face or voice. We focused specifically on biographic data rather than names in our familiarity task. While names, episodic memory and biographic data are related types of person information, in that they could be considered as amodal and more conceptual information than the perceptual representations of faces and voices [19], it may not be appropriate to treat them as equivalent. Some models consider names a subset of biographic data [20], but others place name retrieval at a stage subsequent to biographic information processing [5] or postulate a name-processing module distinct from that for biographic data [21]. Neuropsychological evidence also suggests that name retrieval can be impaired independent of the ability to access biographic information from faces, voices or names [22].

Our goal was to determine whether subjects could learn biographic data better if the latter were presented simultaneously with a face, a voice, or both. In the retrieval (test) phase, we presented biographic data alone, allowing us to ask whether multimodal information during the *encoding* phase had an effect. This is distinct from examining whether multimodal information at the *retrieval* stage is more effective at prompting recall. We included two assessments of retrieval—first, a test of familiarity, and second, a test of more specific identification that involved matching biographic data to a name. As a key control in the second and third experiments, we added conditions with the voice and/or face of a generic narrator rather than the voice and/or face of the specific character whose biographic data were being conveyed. This served as control for any overshadowing effects from divided attention.

## 2. Materials and Methods

### 2.1. Participants

Our study included 31 participants of mean age 24.7 yrs (s.d. 3.9, range 18–33); 27 were female, 4 were male, and 21 were right-handed. All had lived for at least 10 years in a culture with predominant exposure to Caucasian faces. None reported problems with vision or memory, or difficulties remembering faces, voices or names. None had a central neurologic disorder or reported a prior diagnosis of autism. Subjects received CDN $10 for participating. The Institutional Review Boards of the University of British Columbia and Vancouver Hospital approved the study, and all subjects gave informed consent in accordance with the principles of the Declaration of Helsinki. Our subjects were assigned to one of three groups of 10 or 11 subjects; power analyses indicated that to find a facilitative effect of 10% with a power of 0.80 at an alpha level of 0.05, we needed a sample size of 9 per group.

### 2.2. Stimuli

In the interests of ecologic validity, rather than using static images, we created dynamic audiovisual clips of people talking, as was the case in some previous studies [23,24,25]. We used a Canon Rebel Ti3 Camera set up in a room with consistent lighting to film 48 actors (24 females, 24 males). All actors were Caucasian between the ages of 19 and 29 who spoke with a Canadian accent and had no facial tattoos, piercings, glasses or jewelry. The actors were required to shave facial hair and were not permitted to wear heavy makeup. All wore identical black T-shirts, covered their hair with a black toque that we provided and were filmed against the same black background [26]. Actors were filmed for a series of clips, consisting of several long clips of the actor silently looking around the room and a speaking clip in which they introduced themselves as a fictional character, providing, in order, their name, age, place of birth, occupation, hobbies and other interesting biographic information that we provided, all of which were unique to one particular character. These were described from the first-person point of view. Between the filming of each clip, the actors readjusted their shirt and hat, so as to prevent creases and positioning of clothing from providing inadvertent cues to identity. 

Videos were edited for length using Adobe Photoshop CC (2015.0) and rendered at 30 frames per second. Minor variations in zoom or background lighting were minimized as cues by adding the following random elements at the editing stage. Using a random number generator to determine which alteration was applied to each clip, the following edits were made with Photoshop CC: crop from above, crop from below, crop from the right, crop from the left, zoom in, brighten midtones, darken midtones, darken all levels, decrease brightness 30%, increase brightness 30%, increase brightness 60% or no alteration.

Videos were then encoded to MPEG4(Xvid) and sized to 432 × 240 pixels using the AVI tool on Format Factory 3.6.0. The faces spanned about 12.5° of the visual angle in width and 10° in height. Clips were split into their respective audio (.wav) and visual (.xvd) components using the video codec XvidMPEG-4 in Split AVI 1.1. Background noise was removed from the audio component of the clips using Adobe Audition CC (2015.2) by one of two methods. First, a segment of background noise was selected as the noise profile, which was subsequently removed from the entire clip using the Noise Reduction Process tool set to 40 dB. If a sufficiently long segment of background noise alone was not available, the Adaptive Noise Reduction tool was used, using a 10.09 dB noise reduction, a signal threshold of −0.04 dB, a spectral decay rate of 20 ms/60 dB, a broadband preservation of 101 Hz and an FFT size of 512. Sharp clicks due to a nearby machine at the time of filming were present in a few clips and were removed using the Sound Remover Process tool. In order to make the clips compatible with Experiment Builder, the bit rate of all audio files was converted to 176 kbps and 44,100 Hz using Audacity 2.1.2. The final duration of all audio, video and audiovisual clips was 30 s.

A second set of 48 video clips was created. These clips showed one of two generic narrators, a male or a female, describing the name and biographic information of each of the 48 characters. To guard against confusion between characters and narrators, this information was described from the third-person point of view. Additionally, the male narrator described the information for female characters, while the female narrator described that for male characters, to militate against possible gender congruence effects in encoding. While it is not known whether these congruence effects exist in the learning of biographic information, such effects have been shown in associative learning between voices and faces [27]. The duration of these clips matched that of the first set. 

We also created two sets of word stimuli that presented the biographic information in written form. The first set of textboxes consisted of 48 images, which summarized the same biographic information that had been presented by voice in the two sets of video clips above, now in the form of bullet points of 1 to 4 words, with a total of 15 to 20 words per textbox. These were kept very brief to minimize the amount of reading time involved. The name of the character was displayed as well. This set was used in the learning phases of the experiments.

The second set of textboxes was used in the test phases and consisted of 144 images, each of which presented one item of biographic data, which we call a keypoint, written with only 1–5 words. There were three different items of unique biographic data about each of the 48 characters, hence the total of 144.

### 2.3. Experimental Design

**Figure 1:**
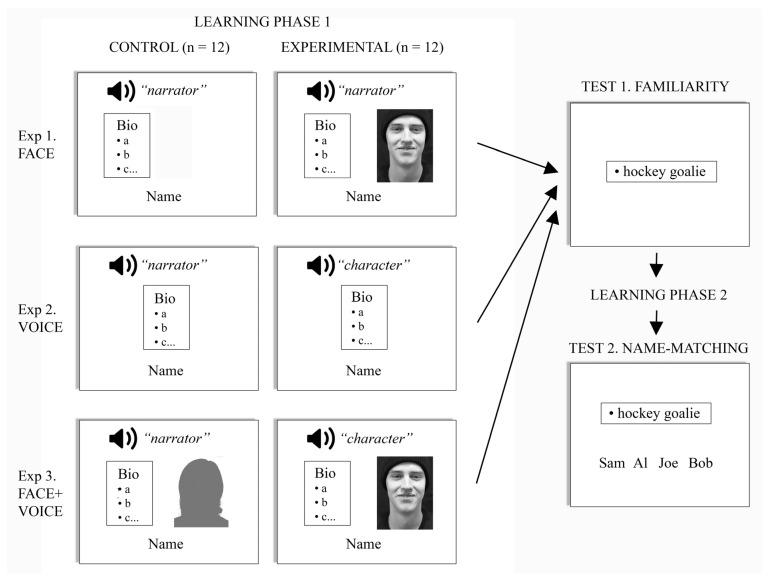
Methods. The learning phases for the three different experiments are shown on the left, with each row showing the control and experimental conditions. Each box depicts the auditory and visual components in each trial. The screen showed the biographic data (’bio’) in brief written format, as bullet points (’•a, •b’, etc., representing these points) in a textbox, with the name of the character below. Audio clips are shown with the speaker symbol; these were voiced by either a narrator or the character, describing the same biographic data for that character. Conditions with video clips are shown with silhouettes or faces; the face shows those conditions with a video clip of the character, while the silhouette represents a generic narrator. After one cycle of learning, subjects had a familiarity test, with a textbox showing one bullet point alone (e.g., ’hockey goalie’). This was followed by the second learning phase of one cycle. The final phase tested identification by having the subject match the single bullet point to the correct name in an array of four choices shown below the textbox.

Our participants were randomly assigned to one of three groups (Figure 1). Each group performed one of three experiments, with each experiment having two conditions that could be compared to answer whether a specific sensory cue facilitated the encoding of biographic information. In each experiment, subjects learned information about 24 target characters, 12 male and 12 female, while the other 24 were used as distractors in the test phase. In the control condition, subjects learned biographic information about the first set of 12 characters. In the experimental condition, the learning phase presented information about the second set of 12 characters along with the face, the voice, or both the face and voice of those characters. Both experimental and control conditions were mixed together in random order in a single learning phase.

We counterbalanced the character sets in two ways to ensure that any effects could not be attributed to specific stimuli. First, for half of the subjects in an experiment, one set of 24 characters was used as targets and the other 24 as distractors, while the sets were reversed for the second half of the subjects. Second, one set of 12 target characters was used for the control condition and the other 12 targets for the experimental condition in half of the subjects, with the set assignment reversed for the rest of the subjects.

Semantic information can be provided either by voice or in written text. Prior studies have used either auditory [24,28,29] or written [19,23,30] means of doing so. It was not clear to us whether these differed in efficacy. Hence, we performed a pilot study in 8 subjects. We found that the accuracy of familiarity judgments about biographic data was better with written than with auditory information (written mean 0.86, s.d. 0.07, auditory mean 0.72, s.d. 0.11, t_(7)_ = 3.43, *p* < 0.01), although the effects on identification accuracy did not differ (written mean 0.60, s.d. 0.14, auditory mean 0.58, s.d. 0.15, t_(7)_ = 0.84, *p* = 0.43). In the end, to ensure uniformity across experiments, we opted to present biographic data in both written and auditory form in each trial.

***Face experiment*.** This was designed to determine whether the encoding of biographic data was enhanced by simultaneous perception of the face of the character being described. In the 12 learning trials of the control condition, the biographic data were shown written in a textbox left of center, while an audio clip of the generic narrator’s voice-over was heard, describing the same biographic information. The name of the character was displayed in the lower screen. The same generic narrator was used for all control trials. In the 12 learning trials of the experimental condition, the audio clip of the same generic narrator’s voice-over was also heard, and the textbox again shown left of center, but now these were accompanied by a silent video clip of the character, to the right of center.

***Voice experiment***. This addressed whether hearing the character’s own voice improved the encoding of their biographic data. The 12 learning trials for the control condition were the same as those in the control condition of the face experiment. That is, biographic information was both shown in a textbox at screen center and also described by an audio clip of the generic narrator’s voice-over. In contrast, the 12 learning trials of the experimental condition replaced the generic narrator’s voice-over with audio clips of the unique voices of each of the 12 characters, describing the same biographic information, but now in the first person. This was again accompanied on the screen by the textbox summarizing the biographic information in written form. 

***Face + Voice experiment***. This final experiment asked whether perceiving both the face and voice of the person enhanced the encoding of biographic data. The 12 learning trials of the control condition presented the textbox as well as an audiovideo clip presenting the face and voice of one of the two generic narrators describing the biographic data for all of the 12 characters. In the 12 learning trials of the experimental condition, the textbox was accompanied by an audiovisual clip of each of the 12 characters describing their own biographic data.

#### Procedure

***Practice run***: Participants performed a short version of the experiment to which they were assigned. This had them learn three characters in the learning phase, followed by the test phase, which probed both the accuracy of familiarity and identification, as below. None of these characters was used in the real experiment. 

***First learning phase***: Participants learned the biographic data about 24 different target characters, as described above for each of the three experiments. They were asked to remember everything they could about the information given. After each of the 24 learning trials, a fixation cross appeared at screen center for 200 msec, and then participants pressed the space bar when they were ready for the next one. If desired, a short break of up to a minute was provided after the 24 learning trials were completed.

***Familiarity test phase***: Participants were shown one item of biographic data (written in 1–5 words), which we call keypoints. There were 72 target keypoints, 3 for each of the 24 target characters, and 72 distractor keypoints, 3 for each of the 24 distractor characters, which differed from the keypoints for the target characters. Each trial presented one keypoint. The 144 trials were presented in random order. The participant made a familiarity judgment by pressing the ‘f’ key for familiar and the ‘u’ key for unfamiliar biographic data. If the participant did not press either key within 4 s, the screen displayed a written message telling them to choose faster, and then, their choice was entered. After the familiarity task was completed, there was again an option for a break of up to a minute. Chance performance on this test would be 50%.

**Second learning phase:** This was a repetition of the first learning phase, though with the order of characters in a new random order. After this second learning phase, there was again an option to take a break of up to a minute.

**Identification test phase:** This consisted of 72 trials, 1 for each of the 3 target keypoints of the 24 characters learned. A keypoint was presented at screen center, while below it were listed four names, side by side, one of which was that of the target character to whom the keypoint referred, and three of which were names randomly selected from the 11 other characters of the same gender who had been seen in the learning phase. The task was to match the keypoint to the correct person by using the mouse to click on the name. Chance performance would be 25%.

In total, the experiment took approximately half an hour to complete. 

### 2.4. Analysis

For the familiarity data, we calculated for the control and experimental conditions of each subject their *accuracy* and *mean response time* for correct trials. The response times of trials that deviated by more than 3 standard deviations from the mean response time for that individual were excluded. For the identification phase, we analyzed only accuracy.

We examined the data of all three experiments together, using a 2 × 3 mixed factor ANOVA, with condition (control, experimental) as a within-subjects factor and experiment (face, voice, face + voice) as a between-subjects factor. Tukey’s HSD test was used to explore significant effects. We performed planned *a priori* comparisons using linear contrasts to determine whether the results differed between the experimental and control conditions of each of the three experiments. This was performed separately for familiarity and identification results. For all statistical tests, the assumptions of ANOVA were not violated unless otherwise indicated. These assumptions were tested using the Shapiro–Wilk test of normality, Levene’s test for homogeneity of variances, Box’s test of equality of covariance’s matrices and Mauchley’s test of sphericity.

## 3. Results

**Figure 2:**
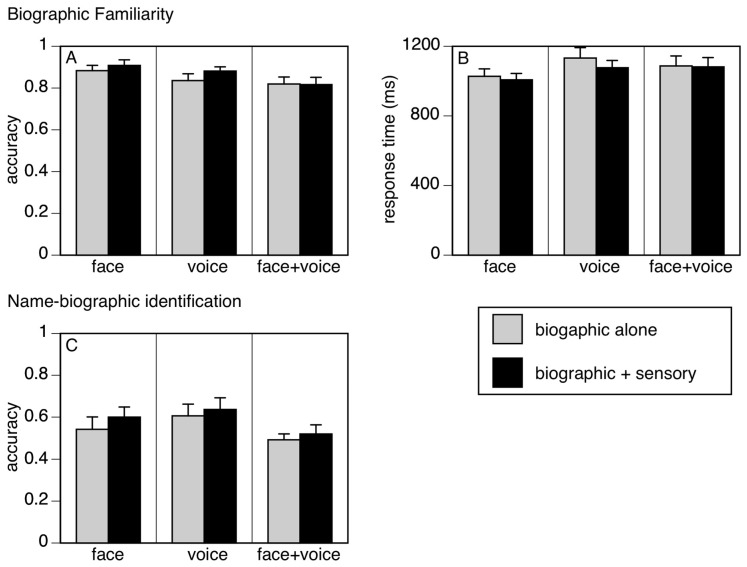
Results. (**A**). Familiarity accuracy, (**B**). Familiarity response time and (**C**). Identification accuracy. Each graph shows results for the three experiments separately (Face, Voice, Face + Voice). The bars compare mean performance of learning the biographic data alone with learning accompanied concurrently with the additional sensory information appropriate for that experiment. Group means are shown, and error bars indicate one standard error.

***Familiarity task.*** For accuracy (Figure 2A), there was a main effect of experiment (F_(1,28)_ = 3.36, I < 0.05, partial η^2^ = 0.002) due to slightly lower accuracy in the *Face + Voice* experiment. There was no main effect of the condition (F_(1,28)_ = 0.91, I = 0.35) or significant interaction between experiment and condition (F_(2,28)_ = 0.35, *p* = 0.70). *A priori* linear contrasts showed no effect of condition in the *Face* (F_(1,28)_ = 0.36 *p* = 0.55), *Voice* (F_(1,28)_ = 1.32, *p* = 0.26) or *Face + Voice* experiments (F_(1,28)_ = 0.004, *p* = 0.95).

For the mean response times of correct familiarity judgments (Figure 2B), there were no main effects of the experiment (F_(2,28)_ = 1.01, *p* = 0.38) or condition (F_(1,28)_ = 1.37, *p* = 0.25) and no interaction (F_(2,28)_ = 0.42, *p* = 0.66). *A priori* linear contrasts showed no effect of condition in the *Face* (F_(1,28)_ = 0.24, *p* = 0.62), *Voice* (F_(1,28)_ = 2.06, *p* = 0.16) or *Face + Voice* experiments (F_(1,28)_ = 0.02, *p* = 0.89).

***Identification task***. This assessed whether subjects could match the biographic data to the correct name. For accuracy (Figure 2C), there was no main effect of the experiment (F_(2,28_) = 1.37, *p* = 0.27) or condition (F_(1,28)_ = 2.30, *p* = 0.14) and no interaction (F_(2,28)_ = 0.14, *p* = 0.87). *A priori* linear contrasts showed no effect of condition in the *Face* (F_(1,28)_ = 1.68, *p* = 0.21), *Voice* (F_(1,28)_ = 0.50, *p* = 0.48) or *Face + Voice* experiments (F_(1,28)_ = 0.38, *p* = 0.54).

Given that our results did not disprove the null hypothesis that there was no difference between the experimental and control conditions, we performed tests for equivalence [31]. These showed that, considering all conditions together, at a significance level of *p* = 0.05, the facilitative effects were not likely to be greater than 0.064 in accuracy or 95 ms in response time for familiarity and no greater than 0.11 in accuracy for identification. 

## 4. Discussion

Although the three experiments were performed by different groups of subjects, the accuracy and response times were equivalent across all three. The results were consistent in showing that adding sensory information about the voice and/or face of the person at the encoding stage did not significantly facilitate the recall of biographic data, either in familiarity or the more specific task of matching the name to biographic data. As this is a null effect, our test of equivalence indicated that the accuracy gains would be unlikely to exceed 0.06 for familiarity or 0.11 for recognition. 

One potential concern is whether the ceiling effects limited our findings on familiarity. However, the mean accuracy in the ‘biographic alone’ conditions was between 81 and 88%, with standard errors of 2 to 3.5%, meaning that the potential improvements should have been discernible. Furthermore, the reaction time data for familiarity also failed to show improvement with additional sensory information, as was the case for accuracy of the name-matching task, which was not at ceiling or floor. 

### 4.1. Interactions between Semantic Information and Face or Voice Learning

How do our results compare with the previous literature? Akin to our study, one report examined the effect of faces and voices on the learning of biographic information [30]). That report had subjects learn a written name and one written item of biographic data about 24 characters, while either seeing a static face (without any sound) or hearing a voice (without any face) reciting part of the Universal Declaration of Human Rights. Learning took place over 18 s for each character. Subjects were better at matching the name to the biographic information when they had seen the face rather than when they had heard the voice. However, there was no condition in which subjects learned the name and biographic information alone, without either the face or the voice. Hence, we do not know whether their results are evidence of facilitation by faces or a detrimental effect of the voices. The latter could reflect an effect of either divided attention for voices (‘voice overshadowing’), which may be due to greater attentional demands for voices, as they are more difficult to process than faces, or linguistic interference between the passage that the voices spoke and the written material on the screen.

In the *reverse* direction, several studies have looked at whether semantic information influences the learning of faces or voices. Four studies examined the effect on familiarity for learned faces. One compared the learning of 20 static faces either alone or with a written name and found better face familiarity with the latter [19]. In another experiment, they obtained similar but more modest results for the written occupation. A second study [29] had subjects learn static faces—20 alone and 20 paired with one of 20 voices that spoke the name and gave one biographic detail about the character; face familiarity was better in the latter condition. However, this study has been criticized by others [24] for using the same pictures of faces at learning and at test, which leaves the possibility that subjects were recognizing images rather than faces. A third study [24] that used similar parameters to ours—i.e., dynamic faces learned over 30 s—showed 36 faces learned alone and another 36 paired with 36 non-synchronized voices, which described the character’s name and several biographic details. They found better familiarity for the faces learned with the semantic data. In these last two studies, however, since the biographic data were given by a unique voice and with a unique name, neither of which were present in the face-only control condition, it is possible that either the name or the voice was responsible for the effect, rather than the biographic data. A final study used a different approach. This had subjects learn 26 dynamic faces while an experimenter narrated the character’s arbitrary name, 13 of which were famous names and 13 of which were not. Subjects had more confident familiarity for the faces learned with a famous name [28]. However, as with the study discussed above [30], there was no control condition with the learning of faces alone. 

There are fewer data on the effect of semantic information on the learning of voices. One study [23] had different groups learn the voice alone, the voice with a dynamic face, the voice with one piece of written biographic data, or the voice with both the face and the biographic data. Apart from finding that the presence of the face at learning led to worse voice familiarity at test, adding biographic data alone neither worsened nor improved voice recognition. Another study tested subjects on voice familiarity before and after associative learning of the voice with either a dynamic face or a name and found that voice familiarity improved by 14% after learning with the face, and by 5% after learning with the name [32]. However, as the study did not include a condition of learning with voice alone, the same issue as in Ref. [30] remains unresolved, namely whether this represents facilitation by the face or interference from the voice.

To summarize, two of the semantic-on-face studies did not isolate the effects of biographic data alone from the effects of names and voices [24,29]. A third showed some improvement of face familiarity if the subject was given the written occupation of the character, though less than that found with names [19]. The fourth showed enhanced face familiarity specific to associations with famous names but did not compare the results to the learning of faces alone [28]. For the semantic-on-voice effect, one showed no impact of biographic data on voice familiarity [23]. In the other direction, effects of face or voice on semantics, which is the topic of our report, there is only one prior study [30]. This showed that remembering biographic data for a name is easier with a concurrent face than with a voice, but we do not know how these results compare to learning biographic data alone.

In the ***Voice*** experiment of our study, the text narrated by the voice duplicated the information shown on the screen; hence, any interference between incongruent written and auditory verbal content was eliminated. Furthermore, both the control and the experimental conditions presented voices—the generic narrator’s in the case of the control and the character’s in the case of the experimental trials. Thus, any effects of divided attention between biographic data and voice stimuli would have been the same in both. Similarly, in the ***Face + Voice*** experiment, the control condition presented the face and voice of the generic narrator, while the face and voice of the character appeared in the experimental trials. With these more equivalent control conditions in place, our study did not find either facilitative or detrimental effects of faces or voices on either familiarity or name matching to biographic data.

### 4.2. Interactions between Face and Voice Learning

Weak or non-existent encoding interactions between biographic and sensory person information do not preclude interactions between different types of sensory cues. Indeed, face/voice interactions may be more likely, given that faces and voices are both sensory cues in person recognition and share a right hemispheric predominance, in contrast to the left hemispheric predominance for verbal information, such as names [8]. This expectation is reflected in models that posit interactions between face and voice recognition units [8,14] and is supported by functional imaging studies that show the coupling of face and voice processing areas during recognition [33]. However, the behavioral evidence for cross-modal modulation of encoding between faces and voices is both mixed and modest, as we now review briefly. 

Two studies examined whether exposure to the voice while subjects learned a static face improved later recognition of the face alone. One study using 60 s of learning found that voices did not improve later recognition of the face in a line-up [17], while another study using 8 s of learning found no difference in an old/new face familiarity task [18]. However, a different approach showed that pairing faces with distinctive voices resulted in better face familiarity than when typical voices were used [34].

More studies have been conducted in the reverse direction, looking at whether faces improve the learning of voices. This perhaps reflects the frequently replicated finding that voices are a weaker cue to person recognition than faces [18,35,36,37] and the expectation from the ’inverse effectiveness principle’ that strong cues are more likely to enhance the performance with weak cues than vice versa [38]. The results have been mixed. Two studies found that voice familiarity was actually poorer rather than better if the voice had been learned with a static face [17,18]. This was attributed to ’face overshadowing’, the capture of attention by the more dominant face cue during encoding. Two studies found no effect on the accuracy of voice familiarity unless the face was presented again in the test phase alongside the target and the distractors [36,39]. One study found that naming the voice was 71% accurate if the voice had been learned with a dynamic face compared to 63% for voices learned alone [40].

One interesting study found that any of these results could be obtained by varying the duration of learning [25]. Showing either static or dynamic faces while the subject learned voices resulted in worse voice familiarity after one to two cycles of learning, each lasting 5840 ms, but no difference after three to four cycles, while dynamic faces enhanced voice familiarity after five to six cycles. This suggested a transition from face overshadowing early on to a facilitative audiovisual integration of dynamic faces and voices that emerges later. However, a review of the studies above does not suggest a simple relationship between the duration of learning and the effect produced. Face overshadowing was seen with 8 s of learning in one study [17] and 60 s in another [17], while learning durations of 30 s in a third study [39] had no effect. Exposure duration is not clearly stated in the one study that found a facilitative effect [40]. Finally, a study that varied learning duration between 6, 20, 60 and 120 s did not replicate the pattern of early overshadowing and later facilitation [36].

In summary, these reports do not show a general consensus on facilitative interactions between face and voice learning. However, several suggest that there may be modifying factors that determine whether facilitation is found, such as the distinctiveness of stimuli [34], the duration of learning [25] and the re-appearance of the associated stimuli at the recall phase [36,39].

### 4.3. Potential Modifying Factors

Our study suggests that when the control conditions make potential general sensory and linguistic effects equivalent to those in the experimental conditions, there is no evidence of facilitation of biographic learning from concurrent sensory representations of the face and/or voice. However, one could ask whether such interactions could be found with conditions or parameters that differ from our experiments. The literature discussed above suggests three possibilities.

First, as suggested by the results of Ref. [25] but not by those of Ref. [36], the temporal dynamics of learning may be critical. Brief periods of learning may show worse performance because of divided attention and overshadowing, which may be mitigated by increasing the duration of learning, allowing facilitative effects to emerge. There are no data on similar modulations by faces or voices on the encoding of biographic data, and cross-modal integration of synchronized sensory information would not explain any facilitative effects on biographic learning. Nevertheless, one could speculate that the learning of biographic data has a similar dynamic. If so, facilitative effects could emerge with more learning time than used in our study. However, the duration of the learning period is comparable between our study and Ref. [25]. They found that facilitation emerged with five to six cycles of learning, a total duration of 29 to 35 s, while in our study, familiarity was tested after one cycle of 30 s of learning, and name matching after a second cycle, or 60 s of learning in total.

A second possibility is suggested by the fact that a number of studies found no difference in voice recognition between voices learned alone or voices learned with faces unless the face re-appeared alongside the voices in the test phase [36,39]. This did not amount to convergence with face recognition because the same faces appeared with both the target and the distractor voices, and thus, the faces were uninformative as to which voice was the target. Rather, this points to facilitative interactions in encoding that require reactivation of those interactions to be manifest. If so, the inference is that the presence of the face at learning does not necessarily enhance voice encoding *per se*, but it establishes a contextual connectivity from face to voice representations, which can later help in the retrieval of voices. This could also explain congruency effects at the retrieval stage—for example, when the voice is more rapidly recognized if presented at test with the face that had been associated with it at the learning phase [26,41], as well as the priming of famous voice familiarity by famous faces [42]. Variations in the strength of associations between various stimuli and biographical data may also explain differences in the retrieval of the latter from faces, names or voices [43,44]. These three are examples of the learning of associations rather than the enhancement of unimodal encoding. One could postulate that having the face or voice presented again at test might similarly enhance the retrieval of biographic data. However, for names at least, similar congruency and priming effects have not been found between names and faces or voices [26,41]. 

A third possibility is that the facilitative interactions are not a general effect but a specific effect of certain stimuli. One study found that familiarity for static faces was enhanced when the face was paired with a distinctive rather than with a typical voice [34]. This was not just an arousal or attentional effect because distinctive sounds did not have the same effect. However, because there was no control condition in which subjects learned faces alone, it is not clear whether there was any effect of the typical voice itself. While we did not choose or evaluate our faces and voices for distinctiveness, it may be that more distinctive stimuli could facilitate the learning of biographic data in a similar manner.

## 5. Conclusions

In summary, we find that, with appropriate design and control conditions to militate against factors such as linguistic interference or divided attention, there is little evidence that the encoding of biographic data is enhanced by concurrent sensory information related to the person, i.e., either the face or the voice. Combined with the results of our companion paper, involving a total of 71 subjects, this shows little evidence that the encoding of semantic or perceptual information benefits from multimodal interactions. Rather, it would be of interest to determine in subsequent experiments whether there is a contextual facilitation of biographic learning by presentation of the face or voice at test, as occurs for the learning of voices [36,39]. If so, this would suggest that, rather than enhancing biographical encoding, associative learning with these cues establishes a connectivity between sensory and biographic data, which can facilitate later retrieval of the latter.

## Data Availability

Data are available in a publicly accessible repository, which does not issue DOIs. They can be found in SFranssondata.xlsx at https://osf.io/ptu6q/files/osfstorage.
